# Purification and Characterization of a Ginsenoside Rb_1_-Hydrolyzing β-Glucosidase from *Aspergillus niger* KCCM 11239

**DOI:** 10.3390/ijms130912140

**Published:** 2012-09-24

**Authors:** Kyung Hoon Chang, Mi Na Jo, Kee-Tae Kim, Hyun-Dong Paik

**Affiliations:** 1 Division of Animal Life Science, Konkuk University, Seoul 143-701, Korea; E-Mails: khchang80@cj.net (K.H.C); ulul55@naver.com (M.N.J.); 2 Bio/Molecular Informatics Center, Konkuk University, Seoul 143-701, Korea; E-Mail: richard44@hanmail.net

**Keywords:** β-glucosidase, *Aspergillus niger* KCCM 11239, ginsenoside Rb_1_, ginsenoside Rd, ginsenoside F_2_

## Abstract

Rb_1_-hydrolyzing β-glucosidase from *Aspergillus niger* KCCM 11239 was studied to develop a bioconversion process for minor ginsenosides. The specific activity of the purified enzyme was 46.5 times greater than that of the crude enzyme. The molecular weight of the native enzyme was estimated to be approximately 123 kDa. The optimal pH of the purified enzyme was pH 4.0, and the enzyme proved highly stable over a pH range of 5.0–10.0. The optimal temperature was 70 °C, and the enzyme became unstable at temperatures above 60 °C. The enzyme was inhibited by Cu^2+^, Mg^2+^, Co^2+^, and acetic acid (10 mM). In the specificity tests, the enzyme was found to be active against ginsenoside Rb_1_, but showed very low levels of activity against Rb_2_, Rc, Rd, Re, and Rg_1_. The enzyme hydrolyzed the 20-C,β-(1→6)-glucoside of ginsenoside Rb_1_ to generate ginsenoside Rd and Rg_3_, and hydrolyzed 3-C,β-(1→2)-glucoside to generate F_2_. The properties of the enzyme indicate that it could be a useful tool in biotransformation applications in the ginseng industry, as well as in the development of novel drug compounds.

## 1. Introduction

β-Glucosidases (β-d-glucoside glucohydrolase, EC 3.2.1.21) are a heterogeneous group of enzymes that are capable of cleaving the β-glucosidic linkages of aryl and alkyl β-glucosides, β-linked oligoglucosides, and several other oligosaccharides, with the release of glucose, generally in the β configuration [1,2]. This enzyme is also commonly referred to as gentiobiase and cellobiase. The enzyme β-glucosidase may be considered to be ubiquitous, occurring widely throughout the bacteria, plant and animal kingdoms, although its functions differ broadly according to the type of organism in which it occurs [3]. β-glucosidase performs important functions in a variety of enzymatic conversion processes, depending on the nature and diversity of the glycone or aglycone moiety of their substrates, since significant differences in the substrate specificity of β-glucosidases have been observed, even when they were derived from the same source [4–7].

The root of *Panax ginseng* C. A. Meyer (Araliaceae) is employed as a traditional medicine, and is used to treat a broad variety of ailments [8]. The pharmacological effects of ginseng originate principally from ginsenosides (ginseng saponins). Thus far, more than 50 types of ginsenosides have been isolated and identified in the roots of *Panax ginseng* C. A. Meyer [9]. The major ginsenosides, including ginsenoside Rb_1_, Rb_2_, and Rc, are in greater abundance in ginseng, whereas the minor ginsenosides, such as ginsenosides Rd, Rg_3_, Rh_2_, and compound K, are present at low levels in ginseng [10]. Recently, several reports have shown that the minor ginsenosides have some remarkable pharmacological activities, and may constitute excellent candidates for use in future drug development. Therefore, many researchers have focused on the conversion of major ginsenosides to minor ginsenosides using acid or alkaline hydrolysis [11,12], heating [13], and enzymatic methods [14]. Among these methods, biocatalytic approaches have become the predominant conversion modality, owing to their marked selectivity, mild reaction conditions, and environmental compatibility.

*Aspergillus* species are one of the most important microorganisms employed in biotechnological techniques for the production of extracellular enzymes. Among *Aspergillus* species, *A. niger* is considered rather attractive to the food industry, since *A. niger* has been recognized as GRAS (generally regarded as safe) by the FDA [15]. In this study, an extracellular β-glucosidase, which specifically hydrolyzes ginsenoside Rb_1,_ was purified from *A. niger* KCCM 11239, and the properties of this purified β-glucosidase were characterized. Furthermore, other by-products after treatment with the enzyme were identified using high-performance liquid chromatography (HPLC).

## 2. Results and Discussion

### 2.1. Growth and β-Glucosidase Production

**Figure 1:**
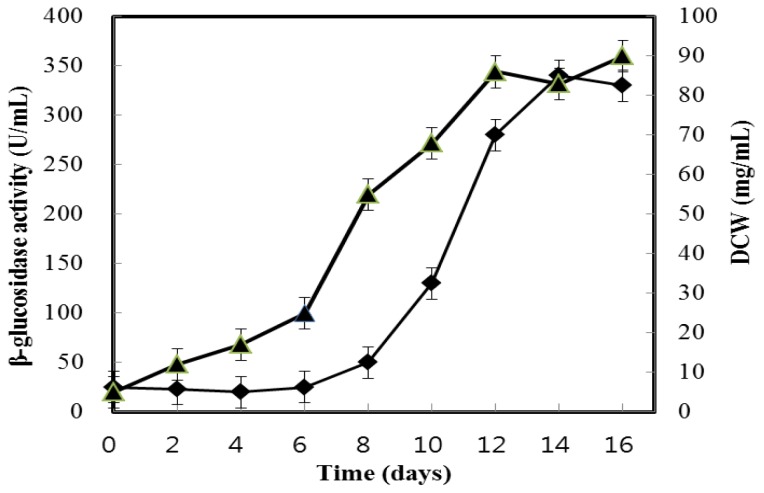
Time course of growth and β-glucosidase production from *Aspergillus niger* KCCM 11239. ■, dry cell weight (DCW); ▲, β-glucosidase activity (U/mL).

Changes in dry cell weight (DCW) and the β-glucosidase activity of *A. niger* KCCM 11239 on PDB medium at 30 °C were assessed under aerobic conditions. Figure 1 illustrates the relationship between β-glucosidase activity for the supernatant and cellular growth. As can be seen in Figure 1, the formation of extracellular β-glucosidase activity is growth-associated, ending when the stationary phase of growth is reached. A maximum β-glucosidase activity of cultivation medium was obtained after 16 days (specific activity, 9.9 U/mg). After that, the enzymatic activities were not changed during further culturing. Cultivation was, therefore, halted at 16 days. The culture supernatants were collected and employed as the source of enzyme purification.

### 2.2. Purification of β-Glucosidase

**Figure 2:**
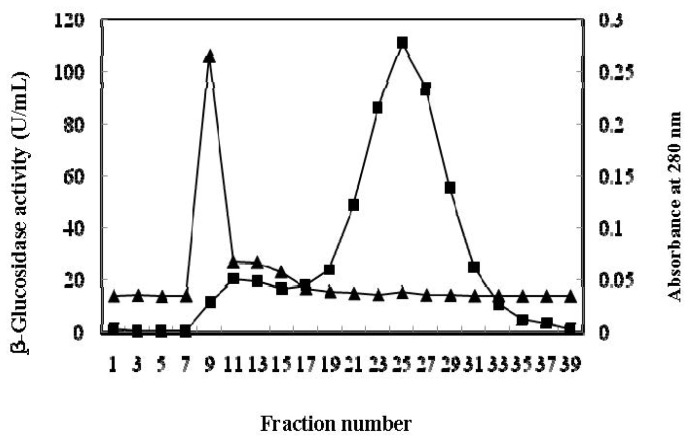
Chromatogram of the crude enzyme on a Sephadex G-100 column. The enzyme was eluted with 0.02 M sodium acetate buffer (pH 4.0). The volume of each fraction was 3 mL. ▲, β-glucosidase activity (U/mL); ■, Absorbance at 280 nm.

**Figure 3:**
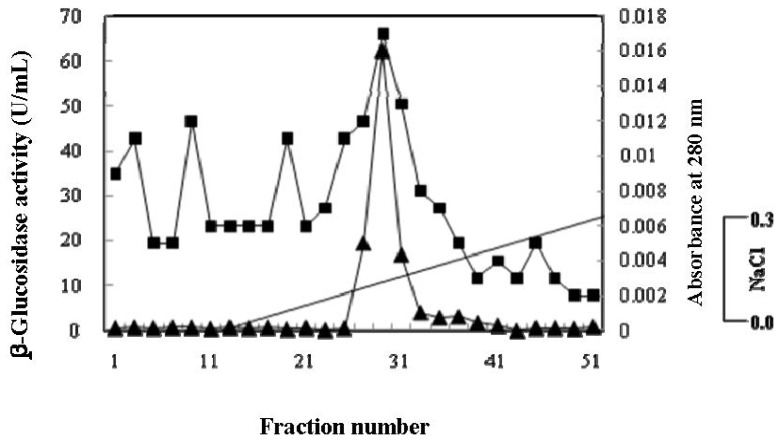
DEAE Sephadex column chromatogram of Sephadex G-100 β-glucosidase fractions. The enzyme was eluted with 0.02 M sodium acetate (pH 4.0) at a flow rate of 1.0 mL/min. The volume of each fraction was 3 mL. ▲, β-glucosidase activity (U/mL); ■, Absorbance at 280 nm.

The β-glucosidase from the culture supernatant of *A. niger* KCCM 11239 was purified using ammonium sulfate precipitation, gel chromatography, and ion-exchange chromatography. The protein in the crude enzyme was precipitated with 30%–90% ammonium sulfate, and was subsequently separated via gel filtration chromatography on a Sephadex G-100 column. The results of β-glucosidase activity and protein from each fraction during the elution of the ammonium precipitate absorbed onto a Sephadex G-100 column are depicted in Figure 2. A fraction volume was 3 mL and an activity measurement revealed only a single peak on the column and major peaks at fraction numbers 8–10. The active fractions were pooled and purified further via anion-exchange chromatography on DEAE-Sephadex. The β-glucosidase activity was observed at fraction numbers 28–29 in 0.15–0.20 M NaCl. The results of β-glucosidase activity and the protein profile on DEAE-Sephadex column are shown in Figure 3. The β-glucosidase was purified by approximately 46.5-fold, with a total volume yield of 1.5% relative to the crude enzyme. The specific activity of the purified enzyme was measured at 460.5 U/mg of protein. The purification steps subsequent to the removal of cells and their results are summarized in Table 1.

### 2.3. Molecular Mass of the Purified Enzyme

**Figure 4:**
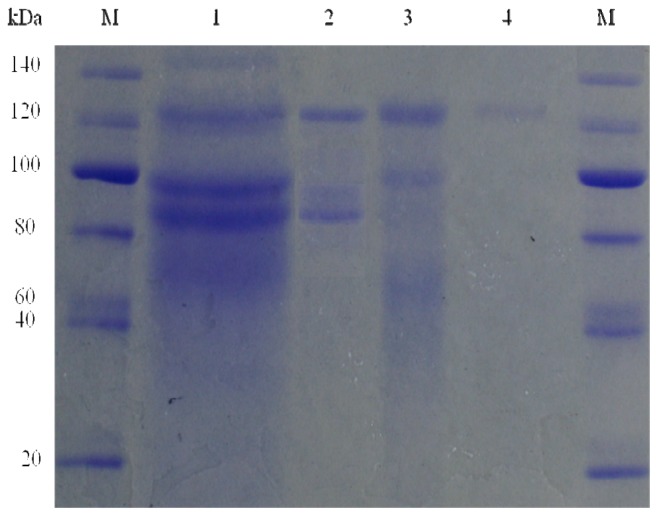
Sodium dodecyl sulfate polyacrylamide gel electrophoresis (SDS-PAGE) of the purified enzyme produced by *Aspergillus niger* KCCM 11239. M, protein marker; lane 1, supernatant fraction; lane 2, ammonium sulphate precipitation (30%–90%); lane 3, Sephadex G-100; lane 4, DEAE Sephadex.

The purified β-glucosidase appeared to be homogeneous according to the criteria of SDS-PAGE, as shown in Figure 4. The molecular weight of the purified β-glucosidase was estimated at approximately 123 kDa, based on calculations of its mobility using standard calibration proteins. The β-glucosidase from *A. niger* KCCM 11239 differs from those of other β-glucosidases generated by *A. niger* reported thus far, which were 46, 110, and 240 kDa [16–18].

### 2.4. Effects of pH and Temperature

The optimal pH of the purified β-glucosidase was studied at the range of pH 3.0–pH 10.0. The optimal pH of the purified β-glucosidase was 4.0. The activity of the enzyme was rapidly reduced at pH values above 7.0. The enzyme proved to be highly stable at pH values of 5.0–10.0 and retained more than 70% of original activity after the incubation of the enzyme for 8 h in buffer solution at pH 4.0 at room temperature (data not shown).

The effects of temperature on enzyme activity were evaluated at the optimal pH of 4.0. In our previous study, it appeared that the β-glucosidase activity was significantly affected at higher temperatures and for a longer time (data not shown). An incubation at above 60 °C for 2 h resulted in a 98% loss of activity. In enzymatic assay at various temperatures for 10 min, the purified enzyme showed maximum activity at a temperature of 70 °C, presuming that the enzyme treated with these conditions was denatured in the direction of increasing the activity. At above 70 °C, the enzymatic activity dramatically decreased. The other β-glucosidase derived from *A. niger* exhibited a similar optimum pH and temperature [17,18].

### 2.5. Effects of Metal Ions and Reagents

The effects of various metal ions and reagents on the purified enzyme activity are shown in Table 2. The enzyme activity was reduced to 79% ± 0.27% by 10 mM Cu^2+^, and slightly inhibited by Mg^2+^ and Co^2+^. By way of contrast, Na^+^ and Ca^2+^ enhanced enzyme activity slightly at the same concentration. It has known that β-glucosidase from *Cladosporium fulvum* was inhibited strongly by Cu^2+^, and another enzyme from *Melanocarpus* sp. MTCC 3922 was also inhibited by Cu^2+^ [10,19]. In addition, it appeared that almost all reagents tested in this study did not inhibit enzyme activity except for acetic acid at 10 mM. On the other hand, an emulsifier, Triton X-100, activated the enzymatic activity although the mechanism has been clearly unknown yet. In particular, a chelating agent, EDTA, did not inhibit enzyme activity, thereby suggesting that metal ions are not essential for enzyme activation [20].

### 2.6. Substrate Specificity and Time Course of Hydrolysis of Ginsenoside Rb_1_

The substrate specificity of glycosidase is a critical factor for industrial biotransformation applications. The substrate specificity of the purified enzyme was determined by assaying the hydrolyzing activity of various ginsenosides: namely, Rb_1_, Rb_2_, Rc, Rd, Re, and Rg_1_. When employing different ginsenosides as substrates, the purified enzyme showed marked high hydrolysis activity on ginsenoside Rb_1_, as shown in Table 3. By way of contrast, the activities on Rb*_2_*, Rc, Rd, and Re were either too low or were not detected. The protopanaxadiol (PPD)-type ginsenosides (Rb_1_, Rb_2_, Rc, and Rd) have identical disaccharide glucose-β-(1→2)-glucose linkages at C-3. Ginsenoside Rb_1_ harbors four β-glucosidic linkages, including a C-3, glucose-β-(1→6)-glucose at C-20. Ginsenoside Rb_2_, Rc, and Rd harbor arabinose-α-(1→6)-glucose, arabinose-α-(1→6)-glucose, and glucose-β-linkages at C-20, respectively. The protopanaxatriol (PPT)-type ginsenosides (Re, Rg1) harbor glucosidic linkages at C-6 and C-20. Ginsenoside Re harbors a rhamnose-α-(1→2)-glucose linkage. The results showed that purified β-glucosidase was highly selective for glucose hydrolysis.

**Figure 5:**
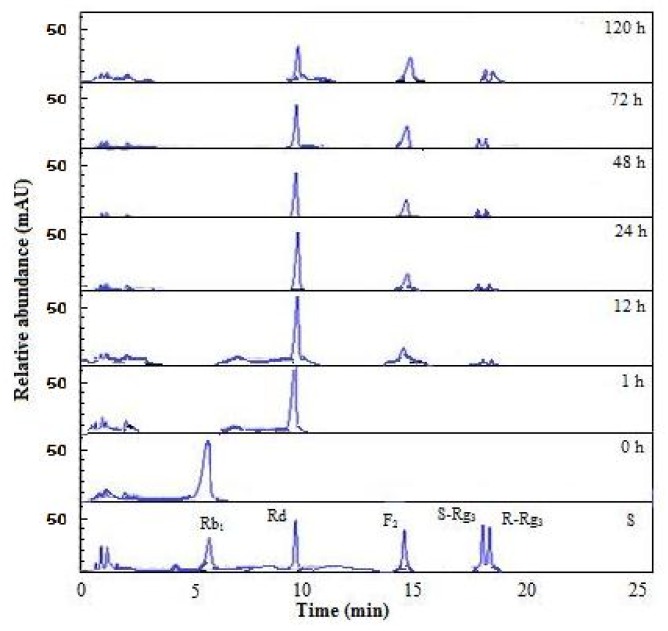
High-performance liquid chromatography (HPLC) analysis of the time-course product of ginsenoside Rb_1_ hydrolyzed by the purified β-glucosidase. S means ginsenoside standards.

To confirm the ginsenoside Rb_1_ enzymatic transformation pathway by β-glucosidase from *A. niger* KCCM 11239, the Rb_1_ ginsenoside was incubated at 50 °C and 200 rpm for 120 h. The hydrolyzed product was periodically assayed via HPLC, as shown in Figure 5. When ginsenoside Rb_1_ was employed as the substrate, four products were identified as ginsenoside Rd, F_2_, S-Rg_3_, and R-Rg_3_.

**Figure 6:**
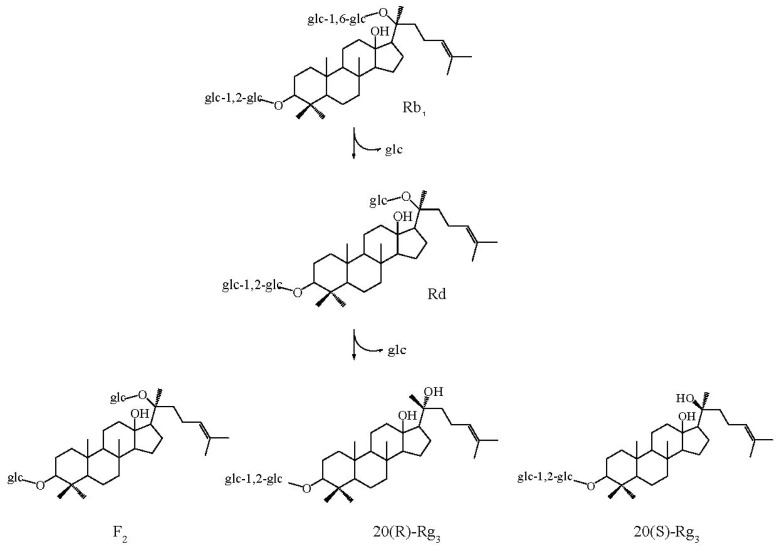
Bioconversion pathway of ginsenoside Rb_1_ by β-glucosidase.

When ginsenoside Rb_1_ was incubated, it was converted completely into Rd after 1 h. After 2 h, the ginsenoside Rd content decreased gradually, and the F_2_ content increased gradually. After 12 h, both S-Rg_3_ and R-Rg_3_ began to be detected, and the content of total Rg_3_ increased over time. This result indicated that the hydrolysis pathway of ginsenoside Rb_1_ by β-glucosidase was Rb_1_→Rd→F_2_ and Rb_1_→Rd→Rg_3_, purified enzyme hydrolyzed a β-(1→6)-glucosidic linkage at C-20, as well as a β-(1→2)-glucosidic linkage at C-3 (Figure 6). These results are similar to those of the pathway by which ginsenoside Rb_1_ is converted to Rg_3_ by *Microbacterium* sp. GS514 [9]. Ginsenoside Rd has been shown to protect neurons against neurotoxic chemicals [21] and to prevent the contraction of blood vessels [22]. F_2_ is a potent inhibitor of acetylcholine-evoked secretion [23]. Rg_3_ induces a variety of pharmacological activities, such as tumor suppression [24], and hepatoprotective [25] and immune-enhancing effects [26]. Therefore, the β-glucosidase obtained from *A. niger* KCCM 11239 is considered a good candidate for use in biotransformation applications for the ginseng industry, and for the development of new drugs.

## 3. Experimental Section

### 3.1. Materials

Ginsenosides Rb_1_, Rc, Re, Rd, Rg_1_, Rg_3_, and Rh_2_, and compound K were purchased from Vitrosys, Inc. (Yeongju, Korea). Sephadex G-100, DEAE Sephadex, bovine serum albumin (BSA), bicinchoninic acid (BCA), and ρ-nitrophenyl-β-d-glucopyranoside (PNPG) were purchased from Sigma-Aldrich (St. Louis, MO, USA). Potato dextrose broth was purchased from Difco (Miller, Becton Dickinson and Co., Sparks, MD, USA). High performance liquid chromatography (HPLC, Agilent 1100 series, Agilent Technologies, Palo Alto, CA, USA) was conducted by using a UV/Vis detector and a gradient pump system. All solvents were of analytical reagent grade.

### 3.2. Growth and Enzyme Production

*A. niger* KCCM 11239 was purchased from the Korean Culture Center of Microorganisms (KCCM, Seoul, Korea). The fungus was grown on potato dextrose agar at 30 °C for 4 days, and the stock culture was maintained at 4 °C. Erlenmeyer flasks were filled to 20% of their volume with potato dextrose broth, and were then inoculated with 5 day-old cultures. The cultures were grown under shaking conditions at 200 rpm for 16 days at 30 °C. During the shake flask culturing, a few glass beads were added to prevent mycelial clumping, and to achieve homogeneous growth. At the specified intervals, the dry weight and enzyme activity of the cells were evaluated.

### 3.3. Assay of Enzyme Activity

β-Glucosidase activity was evaluated via a colorimetric method using PNPG as a substrate. The reaction mixture, which contained 1 mL of 5 mM PNPG and 100 μL of enzyme solution, was incubated for 10 min at 50 °C. The reaction was subsequently terminated via the addition of 1 mL of 0.5 M sodium hydroxide, and the absorption of the released ρ-nitrophenyl (PNP) was measured at 400 nm using a standard calibration curve. One unit (U) of β-glucosidase activity was defined as the quantity of enzyme required to liberate 1 μmole of PNP min^−1^ under standard conditions [27].

### 3.4. Protein Measurement

Protein measurement was conducted with BCA reagent according to the method of Smith *et al.* [28], using bovine serum albumin as a standard protein.

### 3.5. Purification of β-Glucosidase

All steps of β-glucosidase purification were conducted at 4 °C. The crude enzyme from a 16 day-old culture was harvested via 30 min of centrifugation at 8000× *g*, after which the supernatant was precipitated by ammonium sulfate (70%) at 0 °C, and then dissolved in buffer A (20 mM sodium acetate buffer, pH 4.0). The solution was dialyzed exhaustively against the same buffer for 24 h. The dialyzed protein was concentrated and loaded on a Sephadex G-100 column (1.5 cm × 30 cm) equilibrated with buffer A and eluted with 120 mL of the same buffer at a flow rate of 1.0 mL/min. The active fractions were pooled to make a total volume of 44 mL, which was then dialyzed and concentrated prior to ion-exchange chromatography. The active fractions from gel filtration chromatography were applied to a DEAE Sephadex column (3 cm × 15 cm) equilibrated with buffer A. The enzyme was eluted with a linear gradient of 150 mL NaCl from 0 to 0.30 M in the same buffer. The fractions eluted by 0.15 M to 0.20 M NaCl exhibited enzyme activity. The active fractions were pooled, exhaustively dialyzed against buffer A, and were then subjected to molecular weight determinations.

### 3.6. SDS-PAGE

Sodium dodecyl sulfate polyacrylamide gel electrophoresis (SDS-PAGE) was conducted with a 10% gel via Laemmli’s method [29], using a mini-protein system (Bio-Rad). The gels were stained via 0.2% Coomassie’s brilliant blue in a solution containing 45% methanol and 10% acetic acid. The molecular weight was estimated using Protein Size Markers (20–140 kDa) from Elpis Biotech Inc. (Tae-Jeon, Korea).

### 3.7. Assay of Optimal pH and pH Stability

The optimal pH was assessed in a pH range of 2.0–10.0 using the following buffer systems: 100 mM sodium citrate buffer (pH 3.0), 100 mM sodium acetate buffer (pH 4.0–6.0), 100 mM sodium phosphate buffer (pH 7.0–8.0), 100 mM glycine-NaOH buffer (pH 9.0) and 100 mM sodium hydroxide buffer (pH 10.0). The pH stability of the purified enzyme solution (0.5 mg/mL) was measured according to the residual activity following 8 h of pre-incubation of the enzyme in the above buffers at 50 °C.

### 3.8. Assay of Optimal Temperature and Thermal Stability

The optimal temperature was determined by measuring the enzymatic activities with PNPG as a substrate at a range of temperatures from 20 °C to 80 °C in 20 mM sodium acetate buffer (pH 4.0). The thermal stability of the purified enzyme solution (0.5 mg/mL) was evaluated according to the residual activity after 2 h of pre-incubation of the enzyme in a temperature range from 20 °C to 80 °C.

### 3.9. Effects of Metal Ions and Reagents

The effects of metal ions and reagents on β-glucosidase activity were determined via the addition of 10 mM of various divalent (Fe^2+^, Cu^2+^, CO^2+^, Mg^2+^, Zn^2+^, Ca^2+^, and Mn^2+^) and monovalent cations (K^+^, Na^+^), as well as reagents (SDS, EDTA, Triton X-100, glycerol, acetic acid, ethanol, and methanol) to the reaction mixtures prior to 30 min incubation at 25 °C. The enzyme activities were subsequently measured in the presence of the metal ions or reagents under standard assay conditions [30].

### 3.10. Substrate (Ginsenosides) Specificity

Ginsenosides (Rb_1_, Rb_2_, Rc, Re, Rd, and Rg_1_) were dissolved in distilled water to a concentration of 0.5 mg/mL as substrates. The mixture of ginsenosides (200 μL) and purified enzyme (200 μL, 460 U) was incubated for 30 min at 50 °C. The reaction was then halted via the addition of an equal volume of *n*-butanol. After centrifugation, the *n*-butanol phase was evaporated to dryness under vacuum and dissolved in methanol. The amount and pattern of hydrolyzed ginsenosides was detected via HPLC. The injection volume was 20 μL and a reverse phase column was used (C_18_, 4.6 × 150 mm, particle size 5 μm). All peaks were determined according to their absorbance at 203 nm. The mobile phase utilized gradient conditions with solvents A (acetonitrile:water = 100:0) and B (acetonitrile:water = 14:86). The solvent A and B ratios were as follows: [20% A (0 min)]; 20% A (5 min); 30% A (10 min); 30% A (15 min); 60% A (20 min); 60% A (23 min); and 0% A (25 min)], with a 1.2 mL/min flow rate. The amount of product was calculated according to its peak area [31].

### 3.11. Time Course of Hydrolysis of Ginsenoside Rb_1_

Two hundreds microliters of purified β-glucosidase (460 U) was incubated at 50 °C with 5 mL of 100 μM ginsenoside Rb_1_ with shaking at 200 rpm. Aliquots were withdrawn at suitable time intervals (0, 1, 2, 4, 8, 12, 24, 48, 72, 96, and 120 h), and the reaction products were separated with 10 mL of water-saturated *n*-butanol and detected via HPLC under the conditions described above.

## 4. Conclusions

A yield of extracellular β-glucosidase from *A. niger* KCCM 11239 was maximized when the stationary phase of growth was reached. Through several purification steps, the β-glucosidase was purified by approximately 46.5-fold (a specific activity of the enzyme: 460.5 U/mg of protein) and the molecular weight of β-glucosidase was estimated to be approximately 123 kDa. The enzyme activity was reduced to 79% ± 0.27% and 86.00% ± 4.25% by 10 mM of both Cu^2+^ and acetic acid, respectively, but 10 mM of Triton X-100 increased the enzymatic activity as much as 10%. The enzyme had a maximum activity at 70 °C and a range of pH 9.0–10.0. The hydrolysis rate of ginsenoside Rb_1_ to both S-Rg_3_ and R-Rg_3_ was very low. The enzymatic hydrolysis pathway of ginsenoside Rb_1_ was Rb_1_→Rd→F_2_ and Rb_1_→Rd→Rg_3,_ thus suggesting that the purified enzyme hydrolyzed both the β-(1→6)-glucosidic linkage at C-20 and the β-(1→2)-glucosidic linkage at C-3. Rg_3_ has been known to have a potent bio-functional effect on human health. Therefore, β-glucosidase obtained from *A. niger* KCCM 11239 is considered to be worthy of application to the biotransformation technique for the development of new pharmaceutical medicine in the ginseng industry.

## Figures and Tables

**Table 1:** Summary of purification of the β-glucosidase produced by *Aspergillus niger* KCCM 11239.

Purification step	Total activity (U)	Total protein (mg)	Specific activity (U/mg)	Yield (%)	Purification (Fold)
Crude extract (supernatant), 400 mL	86,971.0	8,797.0	9.9	100	1.0
(NH_4_)_2_SO_4_ Precipitation, 86 mL	22,655.0	248.8	91.0	21.5	9.2
Sephadex G-100 Chromatography, 44 mL	3,073.8	13.5	227.1	11.0	22.9
DEAE Sephadex, Chromatography, 6 mL	712.3	1.5	460.5	1.5	46.5

**Table 2:** The effects of metal ions and reagents on the activity of β-glucosidase from *Aspergillus niger* KCCM 11239.

Metal ions or reagents a	Relative activity (%) b
None c	100.00 ± 0.00 d
FeCl_2_	92.00 ± 1.79
CuSO_4_	79.00 ± 0.27
CoCl_2_	88.00 ± 1.50
KCl	97.00 ± 1.45
MnCl_2_	97.00 ± 4.11
ZnSO_4_	94.00 ± 2.76
CaCl_2_	101.00 ± 2.95
MgCl_2_	87.00 ± 8.39
NaCl	101.00 ± 1.29
Ethanol	97.00 ± 3.98
Glycerol	95.00 ± 3.60
Acetic acid	86.00 ± 4.25
Triton X-100	110.00 ± 5.25
Methanol	94.00 ± 2.77
SDS	95.00 ± 1.54
EDTA	97.00 ± 5.00

a The concentration of all chemicals tested in this study was adjusted to10 mM;

b The relative activity was determined via pre-incubation of the purified enzyme with each chemical at 25 °C for 30 min, followed by measurements of residual activity at 50 °C in the presence of the same metal ions and reagents with PNPG;

c The activity against PNPG was considered 100%;

d Results are presented as means ± standard deviations (*n* = 2, *p* < 0.05).

**Table 3:** Relative activities of *Aspergillus niger* KCCM 11239 β-glucosidase on different ginsenosides.

Ginsenoside	Configuration of glycoside linkage	Relative activity (%) a
Rb_1_	−glc(2→1)glc, −glc(6→1)glc	100 ± 0.00 b
Rb_2_	−glc(2→1)glc, −glc(6→1)arap	ND c
Rc	−glc(2→1)glc, −glc(6→1)araf	2.00 ± 0.26
Rd	−glc(2→1)glc	ND
Re	−glc(2→1)rha	ND
Rg_1_	−glc	21.00 ± 0.71

glc: β-d-glucopyranosyl; arap: α-l-arabinopyranosyl; araf: α-l-arabinofuranosyl; rha: α-l-rhamnopyranosyl;

a The enzyme activity on ginsenoside Rb_1_ was defined as 100%;

b Results are presented as means ± standard deviations (*n* = 2);

c Not detected.
